# From Endorsement of Ambivalent Sexism to Psychological IPV Victimization: The Role of Attitudes Supportive of IPV, Legitimating Myths of IPV, and Acceptance of Psychological Aggression

**DOI:** 10.3389/fpsyg.2022.922814

**Published:** 2022-07-07

**Authors:** Vincenza Cinquegrana, Maddalena Marini, Silvia Galdi

**Affiliations:** ^1^Department of Psychology, University of Campania “Luigi Vanvitelli”, Caserta, Italy; ^2^Center for Translational Neurophysiology, Istituto Italiano di Tecnologia, Ferrara, Italy

**Keywords:** ambivalent sexism, attitudes supportive of IPV, domestic violence myth acceptance scale, acceptance of psychological aggression, psychological IPV victimization

## Abstract

Research on intimate partner violence (IPV) has recognized psychological abuse as a precursor of physical and sexual violence in intimate relationships. However, risk factors in predicting women’s psychological abuse victimization in such a context are still unclear. The goal of the present work was to investigate the role of ambivalent sexism on psychological IPV victimization, by taking into account in the same study the effect of three additional social-psychological factors: women’s (i) attitudes supportive of IPV, (ii) endorsement of legitimating myths of IPV, and (iii) acceptance of psychological aggression in intimate relationships. A total of 408 Italian young women (*M*_age_ = 23.87; SD = 2.39) involved in non-marital heterosexual romantic relationships completed measures aimed at assessing (i) hostile and benevolent sexism, (ii) attitudes supportive of IPV, (iii) legitimating myths of IPV, (iv) prevalence of psychological abuse experienced within the last 12 months, and performed a task developed *ad hoc* to measure, and (v) acceptance of psychological aggression in intimate relationships. Results showed that the effect of ambivalent sexism on participants’ prevalence of psychological abuse was mediated by the endorsement of attitudes supportive of IPV and legitimating myths of IPV, as well as by acceptance of psychological aggression. Findings are discussed based on literature about ambivalent sexism, and attitudes and beliefs about IPV.

## Introduction

In 2020, as COVID-19 stormed through UE, many European countries started to report a rise in intimate partner violence [European Emergency Number Association (EENA), 2020; Brink et al., 2021]. Intimate partner violence was already a pervasive social problem before the COVID-19 pandemic, but that has taken on new proportions in the last 2 years [World Health Organization (WHO), 2019]. For example, in 2020, in Italy, during the three-month lockdown, domestic killings accounted for 81% of the total occurred in the entire year (EURES, 2020; Barchielli et al., 2021). This alarming phenomenon is not unique to Italy or UE countries, with the United Nations Entity for Gender Equality and the Empowerment of Women (UN Women) citing restricted movement, social isolation, and economic insecurity due to the COVID-19 pandemic as crucial factors in increasing women’s vulnerability to intimate partner violence around the world (UN WOMEN, 2021).

Intimate partner violence (IPV) encompasses a broad spectrum of acts, ranging from psychological abuse to physical and sexual violence, that occurs in an intimate relationship (Diaz and Hayes, 2012). Compared to physical and sexual violence, psychological abuse, which includes deception, manipulation, humiliation, coercion, intimidation, controlling behaviors (e.g., isolating a person from family and friends, monitoring their movements), as well as threats of physical or sexual violence (Saltzman et al., 2002), is estimated to be the most common form of IPV, with the highest prevalence rates of victimization among young women in intimate heterosexual relationships (Mendoza and Mulford, 2018; Vives-Cases et al., 2021). Psychological abuse is also a precursor of physical and sexual IPV (O’Leary and Smith Slep, 2003; Baker and Stith, 2008; Salis et al., 2014; Cascardi and Avery-Leaf, 2019; Cadely et al., 2020) and may have a unique and sometimes even greater impact on the victim’s health and psychological functioning than physical and sexual attacks typically considered more severe forms of violence (Coker et al., 2000; Mechanic et al., 2008; Follingstad, 2009; Lagdon et al., 2014). Given this evidence, it is imperative to identify factors that increase women’s likelihood of experiencing psychological IPV. However, psychological abuse is one of the dimensions of IPV that has received relatively less attention (Heise et al., 2019).

According to feminist sociocultural perspectives, IPV, in its various forms, is a consequence of gender inequality and is used as a tactic to exert control and dominance over women (Bell and Naugle, 2008). Feminist scholars also argue that (culturally dominant) sexist attitudes strive to perpetuate the subordination and subjugation of women to men, thus representing one of the main sources of violence against women (Dobash and Dobash, 1979; Gelsthorpe and Morris, 1990). However, the relationship between sexist attitudes and psychological IPV victimization for women is conflicting (Forbes et al., 2004; Allen et al., 2009; Alvarez et al., 2021), thus suggesting that additional psychological factors are likely to be at play. These factors include supportive attitudes and understandings of IPV, which have been identified to converge in increasing acceptance of this violence (Carlson and Worden, 2005; Flood and Pease, 2009; McDonnell et al., 2011; Gracia, 2014; del Rio and del Valle, 2017).

Despite plentiful evidence has shown that sexist attitudes affect the extent to which women agree with supportive attitudes towards and beliefs about IPV (Flood and Pease, 2009; Gracia et al., 2020), no study to date has empirically tested the routes from sexist attitudes to psychological IPV victimization by taking into account in the same experiment women’s (i) endorsement of attitudes supportive of IPV, (ii) endorsement of supportive beliefs about IPV, and (iii) acceptance of psychological aggression in intimate relationships. The present study was carried out to fill this gap, by focusing on the (potential) victim making the judgment and her behavior.

Challenging the equation of prejudice with antipathy about sexism, within social psychology the ambivalent sexism theory (Glick and Fiske, 1996, 1999, 2001) represents an important development in the study of sexist attitudes (Glick and Fiske, 2011; Rodríguez-Menés and Safranoff, 2012). According to Glick and Fiske, at the heart of gender relations lies a combination of power difference and intimate interdependence that creates ambivalent attitudes toward women, namely hostile and benevolent sexism. These competitive and subjectively favorable (but patronizing) attitudes toward women, which are common across cultures, influence how individuals perceive and treat women, and serve as complementary ideologies to justify and legitimize traditional gender relations and roles and maintain gender inequality (Sidanius et al., 1994; Glick and Fiske, 1996; Glick et al., 2000; Brandt, 2011).

The ambivalent sexism theory posits that male structural power creates hostile sexism. This derogatory and antipathetic view is directed most strongly at women who do not conform to traditional roles and who allegedly seek to challenge, directly (e.g., feminists, career women) or indirectly (e.g., women who “take advantage” of men sexually), men’s dominant position in intimate relationships or society. In contrast, men’s dependence on women to fulfill domestic roles, sexual and intimacy needs, and to nurture offspring, fosters benevolent sexism. Although directed only toward women who embrace traditional roles (e.g., homemakers), benevolent sexism relies on a gentler and more romanticized view of gender relations. It idealizes women as pure but fragile creatures who ought to be adored, protected, provided for by men, and whose love is required to make a man whole. Therefore, benevolent sexism rewards women with paternalistic affection for “staying in their place,” while hostile sexism punishes women who challenge traditional roles. This view converges with the perception that women who follow conventional and sanctioned roles will be protected and revered by men, and that women who depart from these roles are susceptible to being victims of violence (Lonsway and Fitzgerald, 1995).

Ambivalent sexism has been distinguished as an important determinant of violence against women (Millet, 1970; Gelsthorpe and Morris, 1990; Messerschmidt, 1993; Glick and Fiske, 1996, 2011). For example, IPV and violence against women are more common in those countries and settings where the endorsement of both hostile and benevolent sexism is higher (Brandt, 2011; European Commission, 2016; Zapata-Calvente et al., 2019). Moreover, men who more strongly endorse hostile sexism are more likely to engage in IPV (Reitzel-Jaffe and Wolfe, 2001; Forbes et al., 2004; Hammond and Overall, 2013; Renzetti et al., 2018; Juarros Basterretxea et al., 2019; Martinez-Pecino and Durán, 2019; Ucar and Özdemir, 2021) and violence against women (Viki et al., 2006).

The relationship between women’s endorsement of ambivalent sexism and IPV victimization, however, proves to be not simple: some studies showed no association (Forbes et al., 2004), whereas others found that only hostile sexism (Cantor et al., 2021) or benevolent sexism (Anacona et al., 2017; Vives-Cases et al., 2021) was related to IPV victimization, including psychological abuse victimization. For example, Cantor et al. (2021) showed that hostile sexism predicted psychological abuse experienced by female college students in dating relationships. On the contrary, Vives-Cases et al. (2021) found that adolescent girls with greater benevolent, but not hostile, sexism showed a greater probability of experiencing psychological IPV (see also Anacona et al., 2017). This discrepancy of results suggests that the relation between ambivalent sexism and psychological IPV victimization is not necessarily direct and that additional variables are likely to be at play. For example, this discrepancy in results might be due to the high acceptance of this form of violence (Capezza and Arriaga, 2008), as it is more subtle and ‘invisible’ than physical and sexual IPV (Marshall, 1994, 1996, 1999).

The prompt and accurate recognition of psychologically abusive behaviors between intimates as a form of violence is crucial for victims who need to take action toward changing or leaving the relationship, thus reducing the cumulative harm of the violence (Li et al., 2013; Baldry and Cinquegrana, 2020). However, although women report higher perceived severity of IPV cases (Gracia et al., 2020), studies on the social perception of IPV indicate that this disapproval of violence in intimate relationships coexists with attitudes that are supportive of IPV. That is attitudes that trivialize, tolerate, or minimize the seriousness of the crime, at least in some forms and situations (Waltermaurer, 2012; Gracia et al., 2020).

As a case in point, a small but relevant percentage of both women and men from different countries consider IPV as such only when it involves physical and/or sexual violence or repeated violence (Yamawaki et al., 2009). Specifically, as compared to physical and sexual violence, people tend to judge psychological IPV as ‘not very serious’ and unproblematic (Pipes and LeBov-Keeler, 1997; González and Santana, 2001; Capezza and Arriaga, 2008; Gonzalez-Ortega et al., 2008; Harding and Helweg-Larsen, 2009; Medarić, 2011; Rodríguez-Franco et al., 2012; Larsen and Wobschall, 2016; García-Díaz et al., 2017), sometimes even as a positive occurrence in a relationship (Henton et al., 1983). Women’s attitudes supportive of IPV can therefore bias their perception of it. More importantly, this evidence suggests that when they endorse attitudes supportive of IPV, women would be more likely to undervalue the seriousness of psychological abuse and accept it, thus becoming more vulnerable to experiencing this form of violence.

Attitudes supportive of IPV are generally associated with misconceptions about the nature and meaning of IPV (Sakall, 2001; Yamawaki, 2007; Durán et al., 2010; Masser et al., 2010; Herrera et al., 2014; Vidal-Fernández and Megías, 2014; Giger et al., 2017; Martín-Fernández et al., 2018b; Lelaurain et al., 2019), which play a relevant role in understanding how women may interpret such violence. The literature refers to these misconceptions as domestic violence myths, defined as false but widely and persistently held beliefs that serve to legitimate and justify IPV (Peters, 2008). These include minimizing the occurrence of IPV (e.g., it is a type of violence that does not affect many people), holding the victim responsible for the abuse (e.g., if a woman goes on living with a man who abuses her, then it is to a great extent her responsibility if he abuses her again), and justifying or exonerating the perpetrator (e.g., when a man is violent it is because he lost control of his temper).

In the past 40 years, these legitimating myths of IPV have become less publicly tolerable, at least in Western countries (Fakunmoju et al., 2021). Nonetheless, they are still present. For instance, it has been shown that legitimating myths of IPV are linked to a certain reticence to accept IPV as a reality in some sectors of society, justification of the aggression, and victim responsibility (for example, previous insults, infidelity, going out without permission, etc.), exoneration of the perpetrator, and to nonrecognition of IPV as such (Taylor and Sorenson, 2005; European Commission, 2010; Giger et al., 2017; Cinquegrana et al., 2018; Lelaurain et al., 2018, 2019; Cantor et al., 2021; Fakunmoju et al., 2021). These findings suggest that, to the extent that they endorse legitimating myths of IPV, the likelihood increases for women to legitimate and justify psychologically abusive behaviors. As a result, women will be more likely to accept these abuses perpetrated against them.

Overall, if we are to identify factors that may bias the perception of psychological IPV as acceptable, both attitudes supportive of IPV and legitimating myths of IPV must be taken into account (Carlson and Worden, 2005; Flood and Pease, 2009; Heise, 2011; Waltermaurer, 2012; Gracia and Tomás, 2014; Heise and Fulu, 2014; Gracia et al., 2015; European Commission, 2016; Martín-Fernández et al., 2018a; Copp et al., 2019). As a case in point, Gracia and Herrero analyzed the acceptability of IPV and its correlates in a representative sample of citizens of all member states of the EU. The authors found that higher levels of acceptability were reported by those who blamed women for IPV and perceived IPV as less severe and less frequent. According to this literature, therefore, examining the relations between benevolent and hostile sexism, attitudes supportive of IPV, legitimating myths of IPV, and acceptance of psychological aggression in intimate relationships should provide a more comprehensive framework to clarify the relationship between sexist attitudes and psychological IPV victimization.

## Overview of the Current Study

There is worldwide evidence that both men and women endorse hostile and benevolent sexism, and that women’s endorsement of benevolent sexism is stronger than their endorsement of hostile sexism (Glick et al., 2000; Barreto and Ellemers, 2005; Powell and Webster, 2018). Women endorse benevolent sexism because they view it as relatively harmless (Bosson et al., 2010; Becker and Swim, 2011) or even romantic (Rudman and Heppen, 2003). Moreover, women may feel flattered by offers of protection, cherished by men, or regarded as “the better sex” (Glick and Fiske, 1997). However, the positive veneer of benevolent sexism hides its insidious nature. That is, benevolent sexism not only increases women’s acceptance of their submissive role (Kay and Jost, 2003; Becker and Wright, 2011; Hammond and Sibley, 2011; Connelly and Heesacker, 2012) but also renders hostile sexism more palatable (Napier et al., 2010), such that women’s endorsement of benevolent sexism predicts greater willingness to endorse hostile sexism over time (Sibley et al., 2007). This may be because women who endorse hostile sexism are not hostile against their own gender in-group, but against norm-deviant women who do not match their traditional role conceptions (Becker, 2010).

Drawing from this evidence, the present work investigated the relationship between women’s ambivalent sexism and psychological abuse victimization. Our working model was that, by reinforcing attitudes supportive of IPV and endorsement of legitimating myths of IPV, benevolent and hostile sexist attitudes would increase women’s acceptance of psychological aggression in intimate relationships, which, in turn, would predict vulnerability to experience psychological IPV. To our knowledge, this model has never been empirically tested. Yet it is an important issue to the extent that sexist attitudes are relevant for improving our understanding of women’s psychological IPV victimization.

Specifically, we expected (i) hostile sexism to affect endorsement of attitudes supportive of IPV, and (ii) both benevolent and hostile sexism to predict legitimating myths of IPV. It has long been shown that the conviction with which women adhere to ambivalent sexism favors their acceptance of attitudes supportive of IPV and legitimating myths of IPV (Peters, 2008; Flood and Pease, 2009; Glick and Fiske, 2011; Gracia et al., 2020; Serrano-Montilla et al., 2020). Research has demonstrated that hostile sexism is linked to more lenient attitudes toward the seriousness of offenses committed by men against women and tolerant attitudes toward IPV, as hostile sexism assumes that a victim is exaggerating the seriousness of the incident to gain benefit (such as money or attention) for herself or to dominate or destroy the perpetrator (Sakall, 2001; Herzog, 2007; Martín-Fernández et al., 2018b). Moreover, both hostile and benevolent sexism are related to women’s legitimization of IPV, minimization of its occurrence, exoneration of the perpetrator, and victim blame, as legitimation and justification of IPV contribute to legitimizing gender inequality (Glick et al., 2002; Craig et al., 2006; Yamawaki, 2007; Peters, 2008; Yamawaki et al., 2009; Masser et al., 2010; Valor-Segura et al., 2011; Vidal-Fernández and Megías, 2014; Giger et al., 2017; Lelaurain et al., 2019; Fakunmoju et al., 2021).

In addition, we predicted that the potential effect of hostile and benevolent sexism on psychological IPV victimization would likely also be mediated by the acceptance of psychological aggression in intimate relationships. As discussed in the introduction section, attitudes supportive of IPV and legitimating myths of IPV may play a crucial role in the biased interpretation of psychological IPV. Moreover, among women, past histories of IPV victimization are unrelated to abuse perceptions (Gracia and Herrero, 2006), and those who express acceptance of IPV are more vulnerable to experiencing it (Faramarzi et al., 2005).

## Materials and Methods

### Participants

A total of 408 heterosexual young women volunteered to take part in the study. Participants were Italian citizens, aged between 19 and 30 (*M*_age_ = 23.87; SD = 2.39); 60 (14.7%) were residents of Northern Italy, 61 (15%) residents of Central Italy, and 287 (70.3%) residents of Southern Italy; 333 (81.7%) held a university degree, 74 (18.1%) had a high school degree, and 1 had less than high school education; 359 (88%) were partnered or dating steadily a person (*M*_months_ = 46.93; SD = 34.85), whereas 49 (12%) were single but had been involved in a relationship or had dated someone steadily in the past 12 months. Participants were recruited in the psychology department of the University of Campania “Luigi Vanvitelli.” Those who agreed to participate in the study were also asked to share the link to the study URL with friends and acquaintances who met screening criteria in a snowball procedure. Only heterosexual women, aged between 18 and 30, who had been in a dating or intimate relationship for at least over a month in the past year were eligible for the study. Participants were informed that the study requested to complete an online survey about beliefs and opinions on romantic relationships. Participants provided their informed consent to participate in the study.

### Procedure

The survey was created using the software Surveygizmo and designed in such a way as to avoid any missing data. After consenting to participate in the study, respondents filled out a questionnaire composed of three parts. In Part 1, participants provided socio-demographic information (listed below in the “Measures” section). In Part 2, participants completed three scales aimed at assessing (i) hostile and benevolent sexism (i.e., Ambivalent Sexism Inventory; Glick and Fiske, 1996), (ii) attitudes supportive of psychological and physical violence in relationships (i.e., Intimate Partner Violence Attitude Scale—Revised; Fincham et al., 2008), and (iii) myths that contribute to the legitimation and justification of violence in relationships (i.e., Domestic Violence Myth Acceptance Scale; Peters, 2008). In Part 3, respondents filled out the Measure of Psychologically Abusive Behaviors (MPAB; Follingstad et al., 2015) to determine whether they had experienced some form of psychological abuse by an intimate partner within the last year, and performed a task aimed at assessing how acceptable participants considered a series of behaviors of psychological aggression in an intimate relationship. The order of these three sections of the questionnaire was fixed. At the end of the survey, participants were fully debriefed and thanked for their participation. Before dismissal, they were allowed either to withdraw their data or sign a release form. All participants signed the form. The procedure and materials of the study had been approved by the University Ethics Committee for Psychological Research.

### Materials and Measures

#### Demographics

Participants completed a request for key demographic items: age, gender, sexual orientation, level of education, and relationship status. In the relationship status item, participants were invited to indicate whether they were (a) married, cohabiting, or involved in a committed relationship, (b) dating steadily a person for over a month, (c) single, but had been involved in a relationship, or had dated someone steadily for at least over a month, in the past 12 months, or (d) single and had not dated anyone in the past 12 months.

#### Hostile and Benevolent Sexism

Participants’ hostile and benevolent sexism were measured using the well-known Ambivalent Sexism Inventory (ASI; Glick and Fiske, 1996) translated and validated in Italian by Manganelli Rattazzi, Volpato, and Canova (Manganelli Rattazzi et al., 2008). Structural validity of the Italian version of ASI was supported by both exploratory and confirmatory factor analyses, which confirmed the bifactorial structure originally proposed by Glick and Fiske (1996). Validity of the Italian ASI was further supported by the demonstration that hostile sexism uniquely predicted negative feminine traits attribution and benevolent sexism uniquely predicted positive feminine traits attribution (Manganelli Rattazzi et al., 2008). The ASI includes 11 items related to hostile sexism (e.g., “Women seek to gain power by getting control over men”; “Women exaggerate problems they have at work”) and11 items related to benevolent sexism (e.g., “Women should be cherished and protected by men”; “Every man ought to have a woman whom he adores”). Participants indicated their agreement or disagreement with each item on a 6-point scale ranging from 0 (*strongly disagree*) to 5 (*strongly agree*). Averaged indexes of hostile sexism (Cronbach’s *α* = 0.89) and benevolent sexism (Cronbach’s *α* = 0.87) were calculated such that higher values reflect higher hostile and higher benevolent sexism.

#### Attitudes Supportive of IPV

To assess the degree to which respondents endorsed the use of physical and psychological violence in dating and intimate relationships, the well-known Intimate Partner Violence Attitudes Scale—Revised (IPVAS-R; Fincham et al., 2008) was included in the questionnaire. Given the lack of previous empirical work using the IPVAS-R with Italian participants, the items of the inventory were translated into Italian by an English-Italian bilingual and bicultural specialist. The measure used in the current study was composed of the same 17 items of the original scale, which measures attitudes supportive of IPV in three domains: psychological abuse (Abuse, eight items; e.g., “As long as my partner does not hurt me, ‘threats’ are excused”; “I think it helps our relationship for me to make my partner jealous.” Cronbach’s *α* = 0.72), controlling behaviors (Control, five items; e.g., “It is okay for me to tell my partner not to talk to someone of the opposite sex”; “I think my partner should give me a detailed account of what he did during the day.” Cronbach’s *α* = 0.57), and physical violence (Violence, four items; e.g., “It would not be appropriate to kick, bite, or hit a partner with one’s fist”; “I think it is wrong to ever damage anything that belongs to a partner.” Cronbach’s *α* = 0.74). As with the original measure, participants were instructed to indicate how much they agreed with each item on a scale ranging from 1 (*strongly disagree*) to 5 (*strongly agree*). After reverse-coding the seven items indicating rejection of physical and psychological violence in relationships, internal consistency for the present version of the full IPVAS-R was 0.70 and comparable to that of prior studies (Toplu Demirtaş et al., 2017). An averaged index of overall attitudes toward IPV was therefore calculated such that higher values reflect attitudes supportive of IPV.

#### Legitimating Myths of IPV

Participants’ endorsement of legitimating myths of IPV was assessed using the Domestic Violence Myth Acceptance Scale (DVMAS; Peters, 2008). The DVMAS is an 18-item self-report instrument developed to measure the complex set of cultural beliefs that serve to legitimate and perpetuate violence in dating and intimate relationships. The overall scale has shown internal consistency across different cultural contexts, and good construct and predictive validity, as the DVMAS has been found to correlate with measures of gender-role stereotyping, acceptance of rape myth, sexist attitudes, and gender-specific system justification, as well as to predict perceived IPV victim and perpetrator responsibility (Peters, 2008; European Commission, 2010; Giger et al., 2017). Given the lack of studies using the DVMAS with Italian participants, the items of the original scale (e.g., “Some women unconsciously want their partners to control them”; “When a man is violent, it is because he lost control over his temper”) were translated into Italian by an English-Italian bilingual and bicultural specialist. Participants rated each item on a 7-point scale ranging from 1 (*strongly disagree*) to 7 (*strongly agree*). Estimates of internal consistency of the present Italian version of the DVMAS were satisfactory (Cronbach’s *α* = 0.79) and comparable to that of prior studies (Peters, 2008; European Commission, 2010). Participants’ responses to the 18 items were therefore averaged into a single score of legitimating myths of IPV.

#### Prevalence of Psychological Abuse

To assess whether participants had ever experienced some form of psychological abuse by an intimate partner in the past 12 months, the Measure of Psychologically Abusive Behaviors (MPAB; Follingstad et al., 2015) was translated into Italian and included in the survey. The MPAB is a measure commonly used to identify violations of intimate relationships at the more extreme end of psychological aggression (i.e., abuse), namely behaviors for which recipients believe their partners deliberately intended psychological harm. The scale consists of 14 categories of psychological aggression (i.e., sadistic actions, threatening behavior, isolating, serious manipulation attempts, public humiliation, verbal abuse, wounding one’s attractiveness or sexuality, treating as inferior, creating a hostile environment, monitoring, wounding through threats to fidelity, jealousy, withholding physically and emotionally, and controlling daily actions) that are not overlapping in terms of psychological abuse. Each category consists of three items representing increasingly severe actions (milder, moderate, and severe), with milder actions being not actually mild in nature and only relatively less abusive than moderate or severe level items. Sample items are: “Treated you as useless or stupid as a way to make you feel inferior,” “Threatened to end the relationship as a way to get you to do what he wanted,” and “Yelled and screamed as a way to intimidate you.” According to prior studies on student and non-student samples, the MPAB has shown very good psychometric properties (Follingstad et al., 2015). Participants indicated how often they had experienced each of the 42 behaviors (i.e., 14 categories with three items each) within the last 12 months, on scales ranging from 0 (*never*) to 5 (*almost daily*). In line with Follingstad et al. (2015), a total prevalence score of psychological abuse was then calculated. For each participant, each item listed on the MPAB was recoded as 1 when the respondent reported that the psychologically abusive behavior had been directed toward her in the prior year (regardless of its frequency) and 0 when she indicated having never experienced the behavior in the past 12 months. Thus, the sum score of the prevalence of psychological abuse reflects the number of psychologically abusive behaviors listed on the MPAB that participants experienced in the last year and could range from 0 (none of the behaviors) to 42 (all behaviors).

#### Acceptance of Psychological Aggression

One of the main aims of this work concerned having a better knowledge regarding women’s evaluation and behavioral responses to psychological abusive acts in an intimate relationship. In line with previous studies (Herzog, 2007; Yamawaki et al., 2009; DeHart et al., 2010; Nguyen et al., 2013), therefore, we developed a task, in which respondents were asked to evaluate short hypothetical scenarios. Using the structure of the MPAB (Follingstad et al., 2015) as a model, we selected the five types of psychological abuse that IPV victims experience most frequently [i.e., monitoring, jealousy, verbal abuse, isolating, and creating a hostile environment (Follingstad et al., 1990, 2015; Harned, 2001; Carney and Barner, 2012)]. We then constructed 12 brief scenarios describing daily life episodes of a young woman and her partner (named “S.”), in which participants were required to place themselves in the role of the female protagonist. Each scenario referred to a specific category of psychological abuse (i.e., monitoring: four scenarios; jealousy: three scenarios; verbal abuse: two scenarios; isolating: two scenarios; creating a hostile environment: one scenario) and was worded to incorporate a specific instance of action fitting within the mild or moderate level of the egregiousness of the MPAB. For instance, a scenario dealing with a mild action of jealousy read: “A guy has looked at you and S. has noticed the event. He gets mad at you and accuses you of having looked at that guy intentionally. Then, S. pulls you down and says: ‘I am jealous of you, you are mine’,” whereas a moderate action of isolating was the following “You would like to have more time together, but your spare time does not always coincide. To make more time to spend together, S. wants you to give up some extra activities, such as the gym and going out to see friends.” Given that we were interested in the subjective view of participants, unlike the MPAB, the likely malignant intention of the perpetrator was excluded from all descriptions. Following each scenario, three courses of actions were listed: (a) all in all, it is right what S. did/saw. I continue our relationship (coded 2; acceptable behavior); (b) I do not agree with how S. did/saw it. However, I continue our relationship (coded 1; problematic but acceptable behavior); (c) I break off our relationship (coded 0; unacceptable behavior). For each scenario, participants were instructed to choose the course of action they would perform. Thus, this measure allowed us to derive and combine two types of information that are crucial when assessing the acceptability of an aggressive behavior: (a) whether participants did or did not agree with what S. did/saw and (b) the participants’ response to that behavior. The sum score of acceptance of psychological aggression could range from 0 to 24 (high acceptance of psychological aggression).

#### Scenarios’ Credibility Check

After completing the task, participants were asked to estimate how frequently the actions described in the scenarios may be present in a relationship, using a scale ranging from 1 (*very uncommon*) to 7 (*very frequent*). Respondents also rated how real the actions described in the scenarios were on a scale ranging from 1 (*not at all*) to 7 (*entirely*). Actions were judged frequent in relationships (*M* = 5.02; SD = 1.07; range 2–7) and perceived as real (*M* = 5.84; SD = 1.03; range 4–7), suggesting that a fairly good job was made to construct the scenarios.

## Results

### Descriptive Statistics and Correlations

Descriptive statistics and correlations among study variables (i.e., hostile sexism, benevolent sexism, attitudes supportive of IPV, legitimating myths of IPV, acceptance of psychological aggression, and prevalence of psychological abuse) are presented in Table 1. Overall, in line with past research (Glick et al., 2000; Glick and Fiske, 2001), participants showed greater endorsement of benevolent (*M* = 3.03; SD = 0.99) than hostile sexist attitudes (*M* = 2.56; SD = 0.92) and low agreement on attitudes supportive of IPV (*M* = 1.67; SD = 0.36) and legitimating myths of IPV (*M* = 2.08; SD = 0.63). Looking at responses on the MPAB, we found that 184 participants (45%) reported having never experienced psychologically abuse within the last 12 months, whereas 224 (55%) had experienced at least one of the 42 abusive behaviors. As shown in Table 2, the categories most frequently reported were creating a hostile environment (*n* = 149), verbal abuse (*n* = 111), manipulating (*n* = 99), restriction due to jealousy (*n* = 91), and withhold emotional/physical affection (*n* = 85). With regards to acceptance of psychological aggression, on average, participants accepted one third of the psychologically violent behaviors proposed in the scenarios. Specifically (see Table 3), 78% of women (*n* = 317) considered acceptable monitoring, 60.5% (*n* = 247) jealousy, and 55% isolating (*n* = 226).

**Table 1:** Descriptive statistics and zero-order correlations among study variables (hostile sexism, benevolent sexism, attitudes supportive of IPV, legitimating myths of IPV, acceptance of psychological aggression, prevalence of psychological abuse).

Variables	Correlations					
	*M* (SD)	1	2	3	4	5
1. Hostile sexism	2.56 (0.92)	–				
2. Benevolent sexism	3.03 (0.99)	0.54^***^	–			
3. Attitudes supportive of IPV	1.67 (0.36)	0.35^***^	0.26^***^	–		
4. Legitimating myths of IPV	2.08 (0.63)	0.54^***^	0.41^***^	0.33^***^	–	
5. Acceptance of psychological aggression	4.29 (3.39)	0.24^***^	0.23^***^	0.45^***^	0.30^***^	–
6. Prevalence of psychological abuse	3.34 (5.66)	0.08	0.04	0.21^***^	0.06	0.28^***^

*N* = 408.

*** *p* < 0.001.

**Table 2:** Prevalence of psychological abuse as a function of the 14 categories included in the measure of psychologically abusive behaviors (MPAB).

Categories of psychologically abusive behaviors	*n*	%
1. Sadistic	13	3.2
2. Threats	66	16.2
3. Isolate	32	7.8
4. Manipulate	99	24.3
5. Public humiliation	36	8.8
6. Verbal abuse	111	27.2
7. Wound re: sexuality	36	8.8
8. Treat as inferior	48	11.8
9. Hostile environment	149	36.5
10. Monitor	66	16.2
11. Wound re: fidelity	37	9.1
12. Restriction due to jealousy	91	22.3
13. Withhold emotional/physical affection	85	20.8
14. Control personal decisions	37	9.1

*N* = 408. For each category of psychological abuse, the table shows the number (*n*) and the proportion (%) of participants experiencing at least one behavior in the past 12 months.

**Table 3:** Acceptability of psychological aggression in intimate relationships as a function of the five categories of psychologically abusive behaviors (monitor, restriction due to jealousy, verbal abuse, isolate, hostile environment) included in the acceptance for psychological aggression scenarios.

	Acceptable behavior	Problematic but acceptable behavior	Unacceptable behavior
	Acceptable		Unacceptable
*Monitor*	77.7%		22.3%
He wants to know always where his girlfriend is or what she is doing, because it is a matter of respect	8.4%	33.3%	58.3%
He is upset by the fact that you do not warn him when you go out	12.0%	48.0%	40.0%
He calls you on the phone continuously when you go out for work	7.9%	49.0%	43.1%
He logins to your social network and deletes some of your contacts	0.2%	9.4%	90.4%
*Restriction due to jealousy*	60.5%		39.5%
He asks you for explanations regarding the time you spend with friends and accuses you of hiding something	4.2%	39.5%	56.4%
He gets angry and wrongly accuses you of exchanging looks with guys because of his jealousy	3.4%	21.8%	74.8%
He does not want that other guys get close to you	5.6%	29.2%	65.2%
*Verbal abuse*	8.6%		91.4%
He tells you that he was wrong to trust you and that you are a bad girl just like all women	0.2%	6.4%	93.4%
While he is screaming at you, he shakes you by the arms. He is really hurting you	–	3.7%	96.3%
*Isolate*	55.4%		44.6%
He asks you to give up some extra activities, such as the gym or going out with friends, in order to have more time to spend together	8.8%	45.6%	45.6%
He insults you because you met some friends without him	–	4.4%	95.6%
*Hostile environment*	35.8%		64.2%
He complains in public about what you are wearing	1.7%	34.1%	64.2%

*N* = 408. For each category of the Acceptance for psychological aggression scenarios, the table shows the proportion (%) of participants regard to acceptability/unacceptability of the behaviors proposed.

As expected, hostile and benevolent sexism were correlated, thus supporting the notion that, although hostile and benevolent sexist attitudes are distinct, they both are forms of sexism (Glick and Fiske, 1996, 2001). Moreover, hostile sexism, benevolent sexism, attitudes supportive of IPV, legitimating myths of IPV, and acceptance of psychological aggression were all significantly and positively correlated. No relation emerged between participants’ prevalence of psychological abuse and scores of hostile and benevolent sexism, as well as between legitimating myths of IPV and prevalence of psychological abuse.

### Main Analyses

To test the hypothesized model, a path analysis was conducted using the package Lavaan (Rosseel, 2012) of the software R (R Core Team, 2019). The model was examined including all the variables of interest. Specifically, the prevalence of psychological abuse experienced by participants within the last 12 months was entered into the model as the criterion variable. Hostile and benevolent sexism were entered as predictors, whereas attitudes supportive of IPV and legitimating myths of IPV were modeled as centered mediators, respectively. Indices of acceptance of psychological aggression were included as a centered second order mediator. All paths from the predictors to the criterion variable were estimated, except for the direct paths from hostile and benevolent sexism to the prevalence of psychological abuse. Model adaptation to data was tested using four indices (along with the cut-off values suggested by Hu and Bentler, 1999; indicated in parentheses), namely, the *χ*^2^/df (lower than 3), the CFI (greater than 0.95), the SRMR (equal or smaller than 0.08), and the RMSEA (smaller than 0.06).

**Figure 1:**
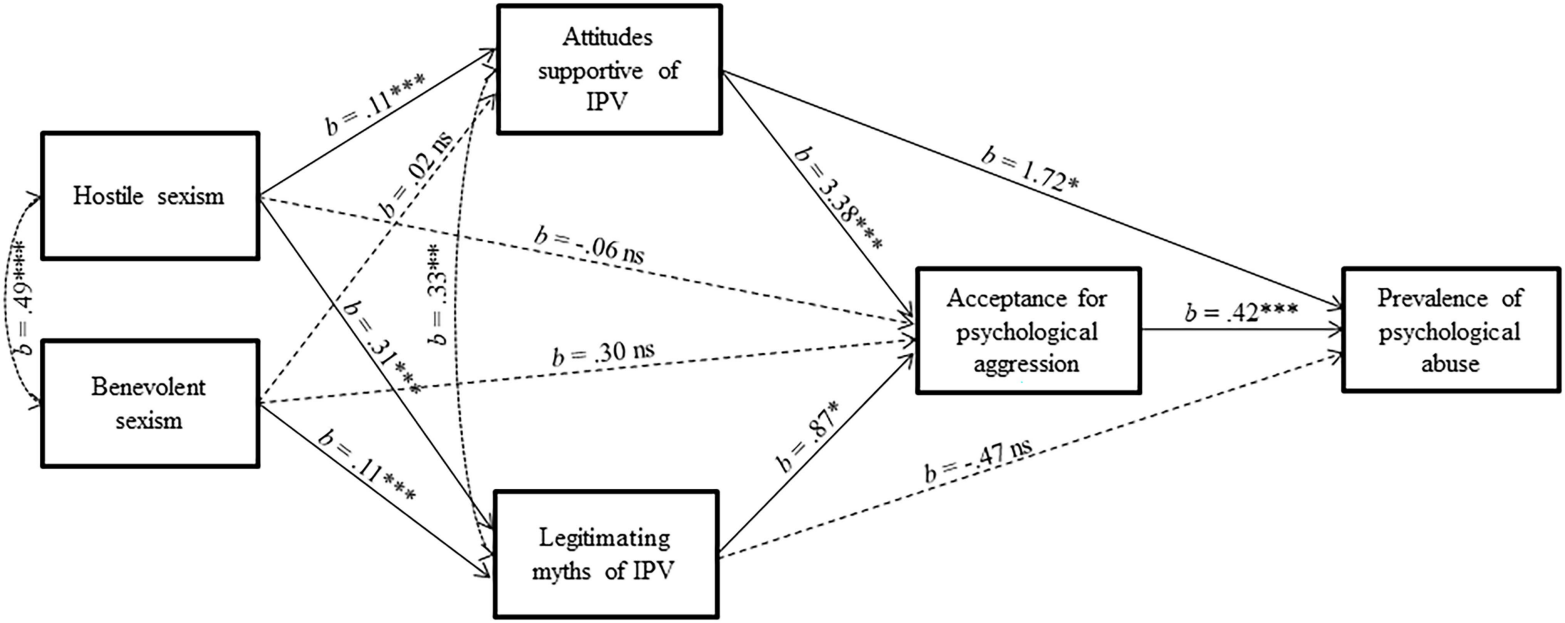
Results of mediation analysis testing the indirect effects of hostile sexism and benevolent sexism on prevalence of psychological abuse *via* attitudes supportive of IPV, legitimating myths of IPV, and acceptance of psychological aggression. *N* = 408. ^***^p < 0.001; ^**^p < 0.01; ^*^p < 0.05.

Results are shown in Figure 1. The proposed model showed a good fit to data, *χ*^2^(2) = 0.41, *p* = 0.82, CFI = 1.00, RMSEA = 0.00 (95% CI = 0.00, 0.06), SRMR = 0.005. As expected, hostile sexism predicted attitudes supportive of IPV, *b* = 0.11, *SE* = 0.022, *z* = 4.974, *p* < 0.001, CI [0.07, 0.15], and legitimating myths of IPV, *b* = 0.31; *SE* = 0.036, *z* = 8.544, *p* < 0.001, CI [0.24, 0.38], but not acceptance for psychological aggression, *b* = −0.06, *p* > 0.78, CI [−0.50, 0.38]. Benevolent sexism predicted legitimating myths of IPV, *b* = 0.11; *SE* = 0.030*, z* = 3.604, *p* < 0.001, CI [0.05, 0.17], but not attitudes supportive of IPV, *b* = 0.03, *p* = 0.191, CI [−0.01, 0.07], and acceptance for psychological aggression, *b* = 0.30, *p* > 0.10, CI [−0.06, 0.65]. Therefore, as hypothesized, higher endorsement of both hostile and benevolent sexism was related to greater endorsement of legitimating myths of IPV. However, hostile, but not benevolent, sexism was related to attitudes supportive of IPV. Importantly, both attitudes supportive of IPV and legitimating myths of IPV predicted scores of the acceptance of psychological aggression (attitudes supportive of IPV: *b* = 3.38; SE = 0.466*, z* = 7.248, *p* < 0.001, CI [2.46, 4.28]; legitimating myths of IPV: *b* = 0.87; SE = 0.344*, z* = 2.520, *p* < 0.01, CI [0.20, 1.55]). Finally, when hostile sexism, benevolent sexism, attitudes supportive of IPV, legitimating myths of IPV, and scores of the acceptance of psychological aggression were entered simultaneously in the model predicting prevalence of psychological abuse experienced in the last 12 months, the effect of the acceptance for psychological aggression was significant, *b* = 0.42; SE = 0.114*, z* = 3.668, *p* < 0.001, CI [0.20, 0.65]. Notably, the direct effect from attitudes supportive of IPV to prevalence of psychological abuse was also significant, *b* = 1.72; SE = 0.878*, z* = 1.958, *p* = 0.05, CI [0.00, 3.51].

To test for the significance of the indirect effects of hostile sexism and benevolent sexism on the prevalence of psychological abuse through the three mediators (attitudes supportive of IPV, legitimating myths of IPV, and acceptance of psychological aggression), we calculated bias-corrected 95% confidence intervals (CIs) using a bootstrapping technique with 5,000 resamples (Preacher and Hayes, 2008). Because the null hypothesis of no mediation states that the indirect effect is zero, the null hypothesis is rejected when the CI does not include zero.

For hostile sexism (see Table 4), the CI for the estimate of the indirect effect on the prevalence of psychological abuse through attitudes supportive of IPV and acceptance of psychological aggression in serial order did not include zero 95% CI [0.06, 0.29]. Noticeably, bootstrap bias corrected CI of the overall mediation index for legitimating myths of IPV, and acceptance of psychological aggression in serial order did not include zero as well, 95% CI [0.02, 0.24]. This result confirmed that a key mechanism why the endorsement of hostile sexism may lead to a higher prevalence of psychological abuse among young women is the fact that hostile sexism promotes positive attitudes toward the use of violence in intimate relationships, as well as the endorsement of legitimating myths of IPV, which, in turn, increase acceptance of psychological aggression.

**Table 4:** Estimations of indirect and total effects.

	*b*	*p*	CI [low; upper]
Indirect effects			
*via Attitudes supportive of IPV*			
Hostile sexism ➔ Acceptance of psychological aggression	0.37	0.001	[0.20; 0.56]
Benevolent sexism ➔ Acceptance of psychological aggression	0.10	0.20	[−0.05; 0.25]
Hostile sexism ➔ Prevalence of psychological abuse	0.19	0.09	[0.00; 0.44]
Benevolent sexism ➔ Prevalence of psychological abuse	0.05	0.28	[−0.03; 0.15]
*via Legitimating Myths of IPV*			
Hostile sexism ➔ Acceptance of psychological aggression	0.27	0.02	[0.06; 0.51]
Benevolent sexism ➔ Acceptance of psychological aggression	0.10	0.03	[0.02; 0.19]
Hostile sexism ➔ Prevalence of psychological abuse	−0.15	0.36	[−0.48; 0.16]
Benevolent sexism ➔ Prevalence of psychological abuse	−0.05	0.39	[−0.19; 0.05]
*via Acceptance of Psychological Aggression*			
Hostile sexism ➔ Prevalence of psychological abuse	−0.03	0.79	[−0.22; 0.17]
Benevolent sexism ➔ Prevalence of psychological abuse	0.12	0.13	[−0.02; 0.30]
Attitudes supportive of IPV ➔ Prevalence of psychological abuse	1.41	0.001	[0.65; 2.31]
Legitimating Myths of IPV ➔ Prevalence of psychological abuse	0.36	0.04	[0.07; 0.74]
*via Attitudes supportive of IPV and Acceptance of Psychological Aggression*			
Hostile sexism ➔ Prevalence of psychological abuse	0.16	0.007	[0.06; 0.29]
Benevolent sexism ➔ Prevalence of psychological abuse	0.04	0.25	[−0.02; 0.12]
*via Legitimating Myths of IPV and Acceptance of Psychological Aggression*			
Hostile sexism ➔ Prevalence of psychological abuse	0.11	0.05	[0.02; 0.24]
Benevolent sexism ➔ Prevalence of psychological abuse	0.04	0.06	[0.01; 0.09]
Total effects			
Attitudes supportive of IPV ➔ Prevalence of psychological abuse	3.13	0.001	[1.59; 4.82]
Legitimating Myths of IPV ➔ Prevalence of psychological abuse	−0.11	0.83	[−1.15; 0.87]
Hostile sexism ➔ Acceptance of psychological aggression	0.58	0.007	[0.16; 1.00]
Benevolent sexism ➔ Acceptance of psychological aggression	0.49	0.01	[0.10; 0.86]

*b* coefficients represent unstandardized value.

For benevolent sexism, the CI for the estimate of the indirect effect on the prevalence of psychological abuse through legitimating myths of IPV and acceptance of psychological aggression did not include zero (95% CI [0.01, 0.09]), whereas the CI for the estimate of the indirect effect on the prevalence of psychological abuse through attitudes supportive of IPV and acceptance of psychological aggression in serial included zero 95% CI [−0.02, 0.12]. Therefore, legitimating myths of IPV (but not attitudes supportive of IPV) and acceptance of psychological aggression in serial mediated the effect of benevolent sexism on the prevalence of psychological abuse.

## Discussion

From psychological to physical forms, violence against women is an endemic problem and occurs in every corner of the world (World Health Organization (WHO), 2019). It can have devasting and long-lasting consequences on the victim’s health and psychological well-being (Dillon et al., 2013; MacGregor et al., 2019; Spencer et al., 2019), ultimately impacting thus communities and society as a whole (Henning and Klesges, 2003). Exacerbating these consequences, women are often subjected to repeated experiences of violence rather than isolated incidents (Tjaden and Thoennes, 2000; Cloitre et al., 2001; Marx et al., 2001; Classen et al., 2005), mostly at the hands of intimate male partners (World Health Organization (WHO), 2019). This phenomenon is of particular concern. Given that abuse in intimate relationships typically begins with fairly subtle controlling and coercive behaviors, rejecting these acts of psychological aggression can be challenging, especially when seen in isolation and/or the malignant intention of the perpetrator is unclear (Capezza et al., 2021).

Given this evidence, the purpose of this study was to contribute from a social psychological perspective to the understanding of factors that may contribute to increasing the likelihood of psychological IPV victimization. Within this framework, we focused on hostile and benevolent sexist attitudes, which allowed us to embrace the personal level and the general views and attitudes that permeate the culture of a country at large.

Two lines of research stimulated and then converged on our work. According to the first one, IPV and violence against women, in general, are more common in those countries and settings where endorsement of hostile and benevolent sexist attitudes is higher (Brandt, 2011). The second line of research shows that, at the individual level, hostile sexist attitudes are related to greater men’s engagement in IPV (Reitzel-Jaffe and Wolfe, 2001; Forbes et al., 2004; Hammond and Overall, 2013; Renzetti et al., 2018; Juarros Basterretxea et al., 2019; Martinez-Pecino and Durán, 2019; Ucar and Özdemir, 2021). However, few studies have examined the role of hostile and benevolent sexist attitudes in IPV victimization for women, with conflicting results (Forbes et al., 2004; Allen et al., 2009; Alvarez et al., 2021).

The current study provides evidence that ambivalent sexist attitudes are associated with psychological IPV victimization. However, for the first time, it also shows that attitudes supportive of IPV and legitimating myths of IPV are critical to understanding those associations. We found that hostile and benevolent sexist attitudes shape attitudes supportive of IPV, and legitimating myths of IPV, which, in turn, bias the perception of psychological aggression in the context of intimate relationships as acceptable, thus increasing the likelihood of psychological IPV victimization.

The data collected are particularly intriguing. Indeed, no direct relationship was found between hostile or benevolent sexism and experienced psychological abuse. Instead, in line with our hypotheses, hostile sexism was the stronger predictor of acceptance of the psychological aggression in intimate relationships, through the mediated effect of both attitudes supportive of IPV and legitimating myths of IPV. Moreover, consistent with past research, participants found benevolent sexism preferable to hostile sexism (Glick et al., 2000); however, benevolent sexism also contributed to the acceptance of psychological aggression *via* the complex set of cultural beliefs that legitimate violence in dating and intimate relationships (i.e., legitimating myths of IPV). Therefore, this evidence shows that when women endorse hostile and benevolent sexist attitudes, they are more likely to legitimate and undervalue the seriousness of psychological abuse, and accept it, thus becoming more vulnerable to experiencing this form of violence.

The present results have important practical implications. In Italy, many efforts at prevention are being made through the media and laws to combat domestic violence (i.e., red code law no. 69/2019). These interventions focus primarily on reporting the existence of maltreatment and the risks involved and offering specific resources for victims of abuse. Our findings suggest that it may be necessary to focus also on the ability of women to recognize psychological maltreatment or abuse. In addition, one reason that women might do not recognize an experience as psychological abuse may be the lack of obvious or visible injury, which may lead them to ignore salient threat cues or not fully process important threat-relevant information in subsequent situations. These situations may thus put women at risk for future victimization (i.e., revictimization; Follingstad and Rogers, 2014). Therefore, prevention programs should educate on the barriers that prevent a woman from successfully recognizing psychological abuse and rejecting its subtle forms to maximize women’s ability to ‘read’ dangerous situations and adopt behaviors accordingly.

On this latter point, it is relevant to highlight that subtle forms of psychological abuse should be legally formalized and recognized in the Italian penal code (not just threats and stalking, art. 612–612 bis) like genuine domestic, physical and sexual violence. These subtle forms of psychological abuse should be detectable through recommendations from qualified and trained people on gender and psychological issues, so that women can have adequate resources to reduce their risk of violence revictimization.

Moreover, eliminating both hostile and benevolent sexism is undoubtedly a daunting challenge. Challenges in reducing sexism include, among others, women’s reliance on men for status and resources, which increases the costs of confronting sexism, and benevolent sexism’s positive stereotypes of women, which make sexist attitudes more appealing, more difficult to recognize, and more difficult to confront. Nonetheless, this study highlights that without a fundamental change in the social attitudes that foster, condone, and perpetuate IPV we will not be able to respond effectively to this problem, by substantially reducing its alarming rates. Therefore, if we are interested in reducing the onset of IPV, a main target for public education initiatives should be ambivalent sexism. To this end, psychoeducational workshops and prevention programs aimed at increasing women’s perceptions of sexism as harmful, have been proven to be effective in reducing endorsement of sexism, and increasing willingness to act for gender equality (Zawadzki et al., 2013; Cundiff et al., 2014; de Lemus et al., 2014), which represents the first step toward reducing violence against women.

There are some potential limitations to this study that should be kept in mind to interpret its findings. For example, it provides only cross-sectional data, which do not allow to attest to any causal links. Therefore, future studies should replicate and strengthen these findings by employing a longitudinal design and examining how acceptance of psychological aggression in intimate relationships and psychological IPV victimization change over time. Moreover, our scores of the acceptance of psychological aggression combined two types of information: (a) whether participants did or did not agree with the psychologically aggressive behavior and (b) the participants’ response to that behavior. Therefore, one could argue that this measure does not allow to draw information about the role of attitudes supportive of IPV and legitimating myths of IPV in predicting a specific category of psychologically aggressive behaviors that is crucial when assessing the acceptability, namely problematic but acceptable behaviors (i.e., participants did not agree with the behavior but continued the relationship).

In this regard, supplementary analyses were conducted only considering these problematic but acceptable behaviors. We found that both attitudes supportive of IPV and legitimating myths of IPV significantly predicted both agreement with the psychologically aggressive behavior [attitudes supportive of IPV: *b* = 0.30, *t*(405) = 6.25, *p* < 0.001; legitimating myths of IPV: *b* = 0.18, *t*(405) = 3.80, *p* < 0.001] and behavioral responses to that behavior [attitudes supportive of IPV: *b* = 0.35, *t*(405) = 7.35, *p* < 0.001; legitimating myths of IPV: *b* = 0.17, *t*(405) = 3.52, *p* < 0.001]. These results confirm our findings showing that both attitudes supportive of IPV and legitimating myths of IPV bias the perception of psychological aggression in the context of intimate relationships as acceptable. Nonetheless, to fully grasp the generality of the phenomena investigated here, we encourage future research to extend our model using both attitudinal and behavioral measures of acceptability.

An additional limitation is that the study was conducted only in Italy, which threatens the generalizability of the results. Therefore, additional investigation of attitudes and beliefs toward IPV and toward women in other national contexts is highly recommended. Despite these limitations, the present findings contribute to our understanding of the predictors of women’s psychological IPV victimization and may inform the design of more effective intervention and prevention strategies.

## Data Availability Statement

The raw data supporting the conclusions of this article will be made available by the authors, without undue reservation.

## Ethics Statement

The studies involving human participants were reviewed and approved by Ethics committee of the Department of Psychology of the University of Campania Luigi Vanvitelli. The patients/participants provided their written informed consent to participate in this study.

## Funding

The publication of the present article has been supported by the Department of Psychology, University of Campania “Luigi Vanvitelli”, Caserta, Italy.

## Author Contributions

All authors listed have made a substantial, direct, and intellectual contribution to the work, and approved it for publication.

## Conflict of Interest

The authors declare that the research was conducted in the absence of any commercial or financial relationships that could be construed as a potential conflict of interest.

## Publisher’s Note

All claims expressed in this article are solely those of the authors and do not necessarily represent those of their affiliated organizations, or those of the publisher, the editors and the reviewers. Any product that may be evaluated in this article, or claim that may be made by its manufacturer, is not guaranteed or endorsed by the publisher.
